# The TAGA Study: A Study of Factors Determining Aortic Diameter in Families at High Risk of Abdominal Aortic Aneurysm Reveal Two New Candidate Genes

**DOI:** 10.3390/jcm9041242

**Published:** 2020-04-24

**Authors:** Olga Peypoch, Ferran Paüls-Vergés, Miquel Vázquez-Santiago, Jaime Dilme, Jose Romero, Jordi Giner, Vicente Plaza, Jose Roman Escudero, Jose Manuel Soria, Mercedes Camacho, Maria Sabater-Lleal

**Affiliations:** 1 Servicios Mancomunados de Angiología, Cirugía Vascular y Endovascular, Hospitales de la Santa Creu i Sant Pau/Dos de Mayo, 08025 Barcelona, Spain; opeypoch@santpau.cat (O.P.); jdilme@santpau.cat (J.D.); Jromeroc@santpau.cat (J.R.); jescuderor@santpau.cat (J.R.E.); 2 Genomics of Complex Diseases, Research Institute of Hospital de la Santa Creu i Sant Pau (IIB Sant Pau), 08041 Barcelona, Spain; fpauls@santpau.cat (F.P.-V.); vazquez.miquel@gmail.com (M.V.-S.); jsoria@santpau.cat (J.M.S.); mcamacho@santpau.cat (M.C.); 3 Universitat Autonoma de Barcelona, (IIB Sant Pau), 08025 Barcelona, Spain; jginer@santpau.cat (J.G.); Vplaza@santpau.cat (V.P.); 4 ISGlobal, Hospital Clínic-Universitat de Barcelona, 08036 Barcelona, Spain; 5 Department of Respiratory Medicine, Hospital de la Santa Creu i Sant Pau, 08025 Barcelona, Spain; 6 Angiology, Vascular Biology and Inflammation Laboratory, Research Institute of Hospital de la Santa Creu i Sant Pau (IIB Sant Pau), 08041 Barcelona, Spain; 7 Cardiovascular Medicine Unit, Department of Medicine, Karolinska Institutet, Center for Molecular Medicine, Karolinska University Hospital, 17176 Stockholm, Sweden

**Keywords:** abdominal aortic diameter, aneurysm, AAA, linkage analysis, genetics, risk factors, Epidermal Growth Factor Receptor, EGFR

## Abstract

A variety of disorders are known to be related with aortic geometry, among them abdominal aortic aneurysm (AAA). This work aims to present the main determinants of abdominal aortic diameter in a new cohort of families at high risk of AAA. The Triple-A Genomic Analysis (TAGA) study comprises 407 individuals related in 12 families. Each family was collected through a proband with AAA. We calculated heritability and genetic correlations between abdominal aortic diameter and clinical parameters. A genome-wide linkage scan was performed based on 4.6 million variants. A predictive model was calculated with conditional forest. Heritability of the abdominal aortic diameter was 34%. Old age, male sex, higher height, weight, creatinine levels in serum, and better lung capacity were the best predictors of aortic diameter. Linkage analyses suggested the implication of *Epidermal Growth Factor Receptor (EGFR)* and *Betacellulin (BTC)* genes with aortic diameter. This is the first study to evaluate genetic components of variation of the aortic diameter in a population of AAA high-risk individuals. These results reveal *EGFR*, a gene that had been previously implicated in AAA, as a determinant of aortic diameter variation in healthy genetically enriched individuals, and might indicate that a common genetic background could determine the diameter of the aorta and future risk of AAA.

## 1. Introduction

The average diameter of the infrarenal abdominal aorta varies according to factors, including age, sex, and body size. It ranges between 12 and 19 mm in women and between 14 and 21 mm in men [1,2]. The increase of the aortic size is continuous during life, and the normal expansion rate is about 1–2 mm/year [3]. A variety of disorders are known to be related with aortic geometry, among them abdominal aortic aneurysm (AAA) formation [4,5].

An AAA is a focal dilatation and weakening of the abdominal aorta and it is the most common type of arterial aneurysm. The disease is characterized by structural deterioration of the vascular wall leading to a progressive dilatation, which ultimately might result in a rupture. Rupture of the aorta, the most common complication, has a mortality rate of 90% [6] being the tenth cause of death in men over 55 years in the industrialized world [7]. The prevalence of AAA is unknown, due to the fact that most AAAs are asymptomatic until they rupture [8], although it has been estimated to be 6% in men and 1.6% in women over 50 years [9].

AAA is a complex disease with genetic and environmental risk factors [10,11,12]. The genetic predisposition to develop an AAA was described for the first time in a 1977 work on three siblings who underwent AAA surgical repair after aneurysm rupture [13]. People with a positive family history, especially in first-degree male relatives, have a higher risk of developing AAA [14,15], and the heritability of AAA has been determined to be over 70% [16,17].

Although the prevalence of the diseases related to the abdominal aortic diameter has increased in the past decades due to lifestyle changes and various risk factors, data are sparse regarding normal aorta diameter in the general population. Until now, there are no studies investigating whether the normal variation of the abdominal aortic diameter is due to genetic or environmental differences and if baseline differences in aortic size might affect the risk of aortic diseases.

If heritable effects are important and baseline ‘normal’ aortic diameter plays a role in the later development of aneurysms, studies are necessary to identify specific genetic markers that determine abdominal aortic size. Conversely, if the environment plays a greater role, an emphasis should be put on lifestyle interventions in order to constrain the development of abdominal aortic diameter related diseases. Therefore, identifying which genetic and environmental factors determine aortic diameter could be essential to ultimately guide clinically actionable decisions for early diagnosis, and provide mechanistic insight into aortic diameter related diseases.

This study is the presentation of a new family cohort of patients with AAA and their relatives: TAGA (Triple A Genomic Analysis). Since all families have been selected through a proband with AAA (they partially share a genetic background), and family history is one of the established risk factors for AAA, these participants can be considered as genetically enriched for AAA genetic risk factors. The overarching goal of the project was to investigate whether higher (but not pathological) measures of the aorta at early ages combined with clinical and genetic parameters are associated with subsequent development of AAA and can potentially be used as predictors of disease. The present work was undertaken to study the heritability and the main determinants of base-line abdominal aortic diameter in healthy high-risk individuals. Future follow-up will help elucidate the role of base-line aortic diameter in the development of future events, as well as determine genetic and environmental risk factors of AAA.

## 2. Methods

### 2.1. Subjects and Study Design

The TAGA study is based on a family-based design and the use of endophenotypes to understand disease. An endophenotype is a trait influenced by some or all of the genes predisposing to an illness [18]. As endophenotypes are measurable in both affected and unaffected individuals, they provide greater statistical power to localize and identify disease-related genes than affection status alone. In the present study, we have used aortic diameter as an endophenotype to study aortic diameter-related diseases, with a particular focus on AAA.

The TAGA study comprises 407 individuals related in 12 families that have been collected at the Hospital de la Santa Creu i Sant Pau, in Barcelona, Spain, through a proband with AAA during the years 2012–2016. Probands from the TAGA study were defined as those with dilation of the abdominal aorta with a diameter higher than 30 millimeters (mm). All probands were adult males between 68 and 88 years old. Patients with thoracic or suprarenal aorta dilation, saccular aneurysms with secondary aneurysmal degeneration, mycotic or infectious as well as patients with spontaneous, iatrogenic and post-surgery pseudoaneurysms were not considered as probands. All family members of the proband were collected, regardless of their AAA status. At the moment of collection, only the proband in each family was diagnosed with an AAA. All families included at least 10 individuals and at least three generations (or at least two generations with eight or more siblings). A detailed table with the characteristics of each family pedigree is shown in Supplementary Table S1.

The study was performed according to the Declaration of Helsinki. All participants gave written informed consent for them and for their minor children. All procedures were approved by the Institutional Review Board at Hospital de la Santa Creu i Sant Pau (Ref. 11/2010). The main aim of the TAGA study was to elucidate genetic mechanisms underlying aortic diameter. No previous genetic studies in relation to AAA had been performed to any of the families.

### 2.2. Clinical Conditions

To determine the clinical conditions in all participants, the following criteria were used: Acute myocardial infarction and angina pectoris were considered by clinical history and admission with clinical symptoms, electrocardiogram changes, or a positive enzymatic curve diagnosed by a cardiologist. Brain-vascular diseases were assessed by history of transient ischemic attack or stroke. Arterial hypertension was assessed by clinical history and use of antihypertensive drugs. Dyslipidemia was assessed by clinical history and use of hypolipidemic drugs. Diabetes mellitus was assessed by clinical history and use of insulin or oral hypoglycemic medications (no distinction was made between type 1 or 2). Neoplasm was assessed by clinical history and radiotherapy or chemotherapy treatment.

### 2.3. Phenotype Determination

A large number of clinical parameters were collected for all participants and are shown in Supplementary Table S2. In brief, we obtained current information on smoking behavior, anthropometric measures, blood cell counts, biochemical parameters, and extensive information on medical history and medication. Smoking was considered positive for all current-smokers and ever-smokers to reflect irreversible effects of smoking on aortic diameter.

#### 2.3.1. Ultrasound Measures

Ultrasound measures of the diameter of femoral, popliteal, and infrarenal aortic arteries were performed in all participants with the ACUSON Antares ultrasound system (Siemens Healthcare Diagnostics, Marburg, Germany). For all measures, the maximum transversal diameter of the artery was reported in mm.

#### 2.3.2. Pulmonary Function

Pulmonary function tests, including forced vital capacity (FVC), forced expiratory volume in 1 s (FEV_1_), and the FEV_1_/FVC ratio, were collected by the spirometer DATOSPIR-600 (Sibelmed, Barcelona, Spain), before and after the administration of a bronchodilatation agent (salbutamol 400 μg for 15 min). Given the high correlation between these measures, only measures before the administration of the bronchodilatation agent were included in the analyses. All tests were performed between March 2012 and December 2016 by the same technician.

### 2.4. Blood Collection

Peripheral blood was obtained by venipuncture, after a 12 h overnight fast, and between 9:00 and 12:00 am. to minimize circadian fluctuation. No process of acute inflammation was registered in at least one month before the onset of the study, none of the participants had taken antiplatelet therapy or non-steroidal anti-inflammatory drugs 15 days prior to blood collection, or corticoids and immunosuppressors one month prior to blood collection. EDTA blood was used for DNA and plasma isolation. DNA was extracted from whole blood using a commercial kit (Wizard Genomic purification kit, Promega (Madison, WI, US), Ref. A1125; Lot number 0000293640).

### 2.5. Genome-Wide Genotyping and Imputation

Genome-wide genotyping was performed using the Infinium Global Screening Array-24 v2.0 from Illumina (San Diego, CA, US) (coverage 665,608 variants). Prior to imputation, we performed several quality control filters as follows: Gender mismatches, sample call rate (>95%), genotyping call rate (>98%), heterozygosity test (median plus/ minus four times the interquartile range), Hardy-Weinberg equilibrium test (*p*-value > 10^−6^), and Minor Allele Frequency (MAF > 1%). We also filtered out ethnic outliers by principal component analysis and removed monomorphic markers. After all filtering, nine individuals were excluded. All pre-imputation quality control was performed using PLINK 1.90. All chromosomes were then imputed to the Haplotype Reference Consortium reference panel using the Michigan server [19]. After imputing, variants with imputation quality <0.3 or MAF <0.002 were excluded. After quality control, 10,734,197 variants and 387 individuals remained. 

### 2.6. Statistical Analyses

#### 2.6.1. Effect of Individual Phenotypes on Aortic Diameter

To assess the effect of different phenotypes on the diameter of the aorta we used a linear mixed model with sex, age, and age^2^ as fixed effects and a random effect that captures the polygenic effect on the diameter of the aorta. The correlations on the random effect due to relatedness were modeled by the expected kinship matrix between relatives. In addition, non-randomness recruitment bias (ascertainment bias) [20] was controlled by conditioning each pedigree to the proband that was used for the ascertainment. This adjustment essentially reduces the bias created by the AAA cases, so that the parameter estimates are valid for the general population. Residuals were rank-normalized to fit a normal distribution (see further details on Supplementary Methods). The effect of smoking was calculated considering only individuals older than 18 (first smoking event in our sample). All analyses were calculated using the R package Solarius [21], as interface of the Sequential Oligogenic Linkage Analysis Routines (SOLAR-ECLIPSE, version 8.1.1) [22], which is specifically designed for the genetic analyses in extended pedigrees.

#### 2.6.2. Heritabilities and Genetic Correlations

Heritability is described as the proportion of total variance of the traits that are attributable to the additive effects of genes. We estimated heritability in every one of the 41 variables using mixed models. We then calculated genetic and environmental correlations of all measured traits with the maximum diameter of the abdominal aorta measured in mm. For both calculations, we used the most parsimonious model for each phenotype, considering age, age^2^, sex, and smoke status as possible covariates. All models included the expected kinship matrix between relatives as a random effect. All analyses were calculated using the R package Solarius [21], as the interface of the Sequential Oligogenic Linkage Analysis Routines (SOLAR-ECLIPSE, version 8.1.1) [22], which is specifically designed for the genetic analyses in extended pedigrees.

#### 2.6.3. Multipoint Linkage Analysis

Linkage analysis is based on genetic recombination and estimates chromosomal regions that are identical by descent (IBD) in pedigrees, that is, sections of DNA that are inherited by a common ancestor and segregate with the phenotype. We estimated a multipoint IBD matrix of our sample using the software IBDLD (version 3.38) [23]. We used Reference Consortium Human Build 38 (GRCh38) of the human genome as a reference for our physical coordinates. Genetic positions were interpolated from DECODE 2019 sex averaged genetic map [24]. Filtering and pruning are described in Supplementary Methods. All analyses were done using the R package Solarius [21,22]. We considered a logarithm of the odds (LOD) score equal to or higher than 3 as a cutoff for significance.

#### 2.6.4. Fine Mapping Association Studies

After quality control, 10,734,197 SNPs imputed to the Haplotype Reference Consortium reference panel [25], and 387 individuals remained. Linear mixed models were used to estimate the association effect of every SNP within the linkage signal on normalized aortic diameter residuals, and adjusting for age, age2, sex, and the kinship matrix. We considered the linkage signal to span 1Mb upstream and downstream the highest LOD score. We also used publicly available summary data from a UK-Biobank genome-wide association study (GWAS) (extracted from http://www.nealelab.is/uk-biobank/) to assess possible associations with aortic aneurysm within the region under the linkage signal. Regional plots were done using Locuszoom [26].

#### 2.6.5. Non-Parametric Predictive Model

We then calculated the prediction models with a conditional forest to assess the best combination of the variables that could predict the aorta diameter, both excluding and including the AAA cases (other arteries were not included in the predictive model) (see Supplementary Methods). All analyses were done using Party package in R [27].

## 3. Results

### 3.1. Sample Description

The database includes data from 407 individuals distributed in 12 families recruited through a proband with AAA. Some clinical and demographic characteristics for one of the probands were not available because of *exitus* at the moment of sample collection. All families had between 14 and 92 individuals in at least three generations, with a similar proportion of males and females per family (the maximum difference was, respectively, 60% to 40%). There were no significant differences in the aorta diameter between families (Supplementary Figure S1), and all cases with AAA (5.6%) were male.

Table 1 shows the demographic data of all individuals included in the study. The mean age of the participants in the study was 38.7 (SD = 22.1, aged between 2 and 88 years). A total of 96 individuals (23.6%) were children and adolescents (under 18 years old). Additionally, 48.2% of the individuals were male, and the average age was similar in both sexes (*p* = 0.95). Among all participants, 20.15% were current smokers, 23.83% were ex-smokers and 56.02% were never-smokers. There was a higher percentage of male smokers compared to women (*p* = 0.003). There was also a higher proportion of men with dyslipidemia (*p* = 0.024). Among women, no case of ischemic heart disease had been reported. The rest of the reported variables were similar in both sexes.

The average arterial diameters in every age interval are reported in Table 2. The measured arterial diameters increased throughout life in both sexes and were significantly smaller in women than men in all age intervals except under 20 years. Males had, on average, 1.81 mm higher aortic diameters than females (*p* < 0.001). The age-related increase in aortic diameter was more pronounced in males than in females (0.25 versus 0.2 mm/year, analysis adjusted for the ascertainment effect of probands). The increase was linear throughout life in men, whereas it seemed to reach a plateau in women over 40 years old.

### 3.2. Association between Aortic Diameter and Related Phenotypes

We first performed bivariate analysis between aortic diameter and a total of 41 clinical variables (Supplementary Table S3), adjusting for age, age^2^, and sex. Phenotypes that showed significant association with aortic diameter were considered at false discovery rate <0.01 (Table 3). Our results showed that the most associated phenotypes to “healthy” diameter of the aorta were the diameter of femoral arteries, followed by height, weight, pulmonary function tests, popliteal arteries diameter, levels of serum creatinine, waist circumference, body mass index, and the erythrocyte mean corpuscular volume. The diameter of left arteries (popliteal and femoral) had a tendency to show stronger associations with the aorta diameter than those of the right arteries. All anthropometric measures were highly correlated with each other (data not shown) and indicated a significant positive correlation between larger anthropometric measures and larger diameter of all arteries.

Given the importance of sex, we repeated all bivariate analyses using male-only, however, no significant differences were observed in the results, and no additional significant variables emerged in the sex-stratified analyses.

**Figure 1:**
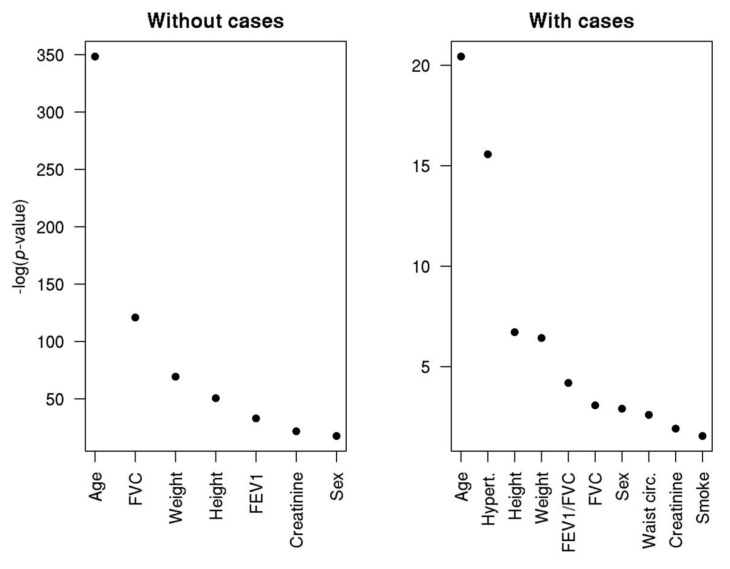
Best predictor variables for aortic diameter. *Y*-axis reports the estimated probability that each variable improves the accuracy of the predictive model.

We then created multivariate predictive models to estimate the diameter of the aorta using all measured variables (except the diameter of other arteries) at the same time to allow the model to select the best estimates of aortic diameter. Results showed that the best predictor of aorta diameter was, by far, age (Figure 1), both in models including or excluding AAA patients from the analysis.

The model to predict aortic diameter in the sample excluding AAA patients was composed of age, height, weight, serum creatinine levels, sex, pulmonary function tests (spirometry), together accounting for approximately 73% of the variability in aortic diameter (51% after including AAA cases). The best model to predict aortic diameter when considering AAA cases additionally included hypertension, waist circumference, and smoking.

Moreover, our results suggested significant interactions between sex and age and also between sex and weight, suggesting that both age and weight have a significantly different effect on the diameter of the aorta in males than in females.

**Figure 2:**
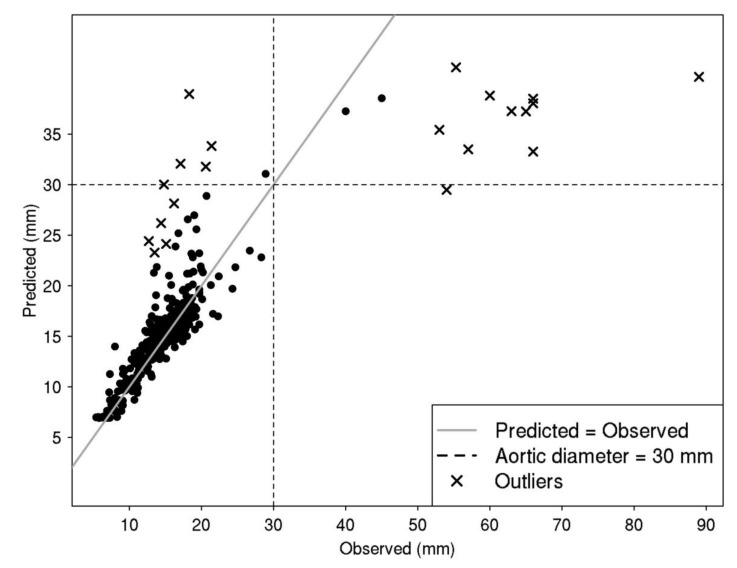
Prediction accuracy of the aortic diameter using the best model.

Using all selected variables, our best model was able to predict the aorta diameter in “healthy” individuals but was unable to predict individuals with an AAA (Figure 2) indicating that additional factors trigger the pathological widening of the aorta, beyond the factors that regulate normal variation of the diameter in healthy individuals.

### 3.3. Heritability and Genetic Correlations

We used linear mixed models to calculate the proportion of the variability explained by genetic factors, based on the information of the pedigrees. Genetic factors (i.e., heritability) explained 0.34 (CI: 0.30–0.39) of the total phenotypic variance in abdominal aortic diameter, after controlling for sex, age, and age^2^. Heritability of the aortic diameter when considering only male participants increased to 0.42 (CI: 0.36–0.50). Heritability of the popliteal arteries ranged from 0.38 (CI: 0.34–0.42) to 0.45 (CI: 0.41–0.49), whereas heritability of the femoral arteries ranged from 0.36 (CI: 0.32–0.40) to 0.41 (CI: 0.37–0.44).

Heritability of other measured phenotypes in the study is shown in Supplementary Table S4.

Linear mixed models can also be used to split global phenotypic correlations into genetic and environmental correlations based on the structure of the pedigrees. This allows discovering common genes determining the variation of aortic diameter and related phenotypes. The most significant genetic correlations are shown in Table 4.

Our results indicated that aortic, femoral, and popliteal arteries are highly genetically correlated, suggesting that a common genetic background determines the diameter of these arteries.

Moreover, other traits exhibited significant genetic correlations with the aortic diameter. The most significant were height and *FEV*_1_, (Table 4), suggesting the existence of common genes that determine pulmonary capacity and the aortic diameter.

Even though serum creatinine showed a strong association with the aortic diameter in the regression analysis and the predictive models, partition of variances showed that this was mainly due to environmental correlation rather than genetic correlation (Supplementary Table S4), and therefore, did not support the hypothesis of common genetic factors underlying creatinine levels and aortic diameter.

### 3.4. Genome-Wide Linkage Analysis Reveals Two Significant Regions Associated with the Diameter of the Aorta

**Figure 3:**
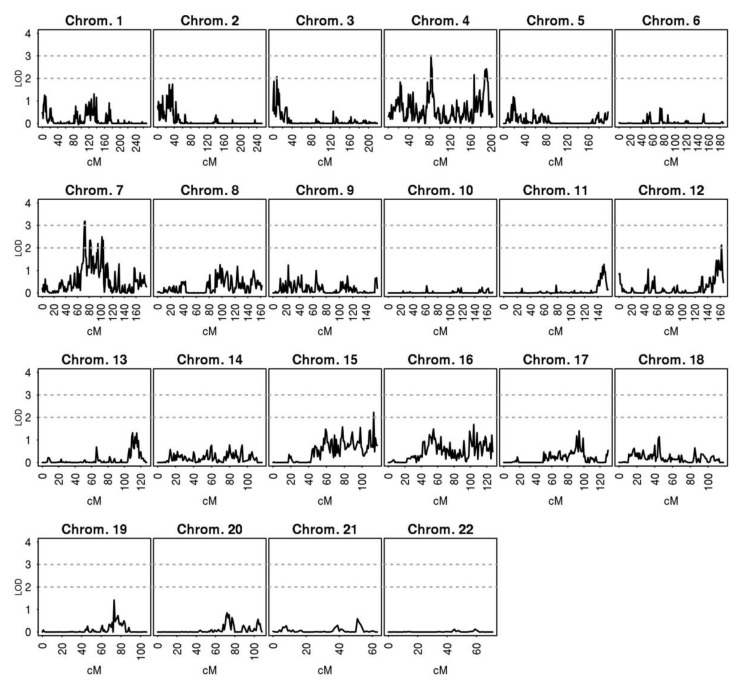
Results of the multipoint genome-wide linkage analyses showing LOD scores on all chromosomes.

**Figure 4:**
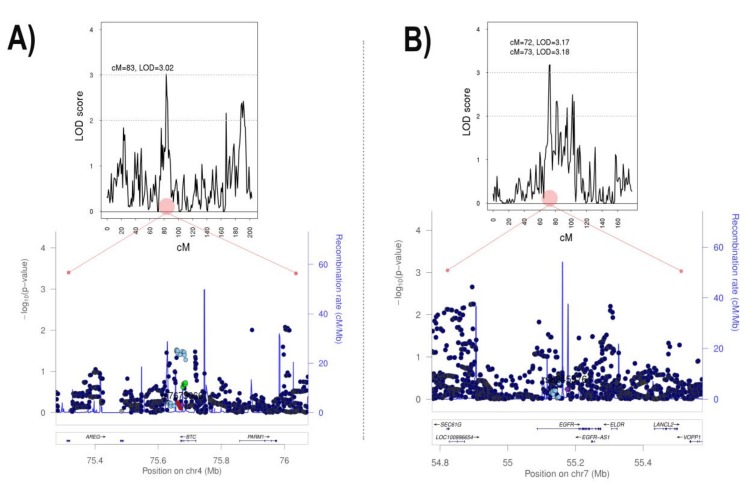
Significant linkage regions on chromosomes 4 (**A**) and 7 (**B**) and regional plots showing association analyses on the region under the maximum LOD score.

Results of a genome-wide multipoint linkage analysis performed at one centimorgan (cM) resolution are shown in Table 5 and Figure 3. Two regions, one on chromosome 4 and one on chromosome 7, showed significant linkage with aortic diameter (maximum LOD score = 3.02, at cM 83 and LOD score = 3.18, at cM 73 respectively, Figure 4). The closest gene/s to the cM with maximum LOD scores were *Endothelial Growth Factor Receptor (EGFR),* and *Betacellulin (BTC)* (Table 5). Despite the increased heritability of the male-only analyses, no significant linkage signals were observed when only males were analyzed (data not shown).

Fine-mapping of the significantly associated region on chromosome 7 was performed using association analysis between 19,989 available imputed SNPs (between physical coordinates 51,752,488 and 67,013,180 (build 38)) and the aortic diameter. Fine-mapping of the significantly associated region on chromosome 4 was performed using association analysis between 9850 available imputed SNPs (between physical coordinates 70,662,849 and 76,845,518 (build 38)) and the aortic diameter. No significant association was found for any SNP within the two linkage regions and aortic diameter in our population (Figure 4), or in a GWAS on aneurysm from UK-Biobank.

## 4. Discussion

This study evaluates the heritability and the genetic basis underlying the diameter of the aorta in a population of unaffected AAA high-risk individuals. It constitutes the first genetic study to evaluate heritability and genetic determinants of normal variation in aortic diameter in extended families. For this purpose, we have used the TAGA study cohort, which constitutes a new collection of high-risk individuals related in families that have been ascertained through a proband with AAA. TAGA was designed as a longitudinal cohort study, where future follow-up will be used to evaluate if aortic diameter at different age intervals and the main phenotypes associated with its enlargement can be used as predictors for AAA. In the present work, we show baseline characteristics of the cohort. Overall, our results suggest the existence of common genetic factors determining the diameter of aortic, femoral, and popliteal arteries, along with body size and pulmonary capacity. More importantly, our results reveal two loci with significant linkage with aortic diameter and identify candidate genes that have previously been associated with AAA, therefore, providing a link between normal variation of aortic diameter and disease.

### 4.1. Normal Variation in the Diameter of the Main Arteries is Regulated by Common Genetic Factors and are Predicted by Age, Sex, Anthropometric Measures, Pulmonary Function Tests, and Creatinine Levels

We calculated heritabilities and the main genetic correlations of the aortic diameter with other clinical and anthropometric parameters. We observed that variation in the diameter of healthy abdominal aorta is strongly determined by genetic factors (34%), which is in agreement with a previous twin study that estimated heritability of abdominal aorta diameter is 40% [28]. Moreover, we show that diameter in abdominal aorta, femoral and popliteal arteries are highly genetically correlated, indicating that common genes simultaneously determine the diameter of the main arteries in the healthy population.

Our study confirms consistent larger aortic diameters in men than in women at all age intervals except at interval below 20 years old. In line with our results, other studies focusing either in children or adults have reported that the diameter of the abdominal (children [29]), and thoracic (adults [30,31]) aorta increases gradually with aging for both genders among all ethnicities and that the diameter of the abdominal aorta is wider in men than in women, both in healthy individuals or in patients with AAA ([1,29,32,33]). While previous studies focused on limited age intervals, this is the first study to evaluate such a global age range in the same population.

Interestingly, our results also show an interaction between age and sex, resulting in a higher growth rate in males at the age interval over 60 years old. This could reflect the additional enlargement at elder ages caused by vascular aging, which can be caused by impaired ability of the arteries to resist oxidative stress and inflammation and result in increased diameter. Whether this increased growth rate in older men could explain the higher risk of AAA in men deserves further investigation.

The importance of body size, which is reflected in the association with anthropometric measures in our study, has also been reported before [2,31,34,35]. Moreover, our genetic analyses reveal strong genetic correlations between some of these variables (especially height) and aortic diameter, which indicate that the relationship between these parameters is explained by the existence of common genes regulating the variability of body size and the diameter of the main arteries.

Interestingly, spirometry lung function tests, including FVC, FEV_1_, and the FEV_1_/FVC ratio, appeared amongst the best predictors of aortic diameter in our cohort, both including and excluding AAA cases, indicating again that lung capacity might be associated with both healthy and pathological aortic diameter. Moreover, genetic analysis showed a strong genetic correlation between pulmonary function tests and aortic diameter, which suggests the presence of common genetic pathways underlying both phenotypes.

Among the most commonly reported co-morbidities of AAA is a chronic obstructive pulmonary disease (COPD) [36], which is a progressive condition caused by long-term exposure to smoke gases and particles, in particular tobacco smoke, and characterized by impaired airflow to the lungs. COPD is mainly evidenced by poor spirometry results [37] and represents a common cause of death worldwide [10]. Despite the strong co-morbidity between COPD and AAA, suggested to be partially caused by a common systemic inflammatory status probably exacerbated by smoking consumption [38,39,40,41], this is to the best of our knowledge, the first time that an association between measures of the pulmonary function obtained through spirometry and “healthy” aortic diameter has been described. Although this association could be partly explained by the established association of lung function with adult height [42,43], our predictive multivariate model suggests an additional effect of the pulmonary function beyond height that needs further elucidation.

We also observed that serum creatinine levels appeared amongst the best predictors of the aortic diameter in the “healthy” population and also upon the inclusion of AAA cases. Creatinine is a chemical waste product in the blood that is produced by muscle and filtered in the kidneys. Levels of creatinine in blood are a reflection of kidney function, and also amount of muscle. Our variance component analyses suggested that the correlation between creatinine levels and aortic diameter was not caused by common genes. These results might indicate that creatinine levels could be reflecting the amount of muscle and body size, which is, as we have seen before, highly correlated with aortic diameter.

Smoking and hypertension are strong risk factors for AAA [44,45,46]. The characteristics of our cohort do not allow us to infer good predictors of AAA before the follow-up data are analyzed. However, we can confirm that, despite the widely reported effect on AAA, a predictive model including age, sex, and anthropometric measures, but not smoking and hypertension, was able to accurately predict aortic diameter in all healthy individuals, suggesting that these parameters do not significantly influence healthy aortic diameter but are relevant determinants of the pathological expansion of the aneurysm, as extensively described.

In this regard, one still unresolved issue in AAA diagnosis is whether variation within the normal range of aortic diameter is associated with AAA risk, or whether the biological mechanisms underlying variation in the diameter of the aorta of healthy individuals is independent of the biological mechanisms underlying the development of an aneurysm. Our observations agree with the hypothesis that the pathological expansion of AAA is regulated, at least in part, by different mechanisms than those regulating variation of the aortic diameter within the normal range. However, it is plausible that baseline aortic diameter could reflect a different susceptibility of some individuals to certain triggers. Previous studies from us and others suggest that these pathological mechanisms could respond to a chronic inflammatory process, including cell infiltration and degradation of the extracellular matrix and vascular structures where smoking or hypertension could exacerbate the process.

### 4.2. Linkage Analysis Reveals a Linkage Region Associated with Aortic Diameter on Chromosome 7

To the best of our knowledge, this is the first genome-wide linkage analysis on the diameter of the aorta, which reveals two significant linkage signals, on chromosomes 4 and 7, determining the variation of aortic diameter in our family cohort. The closest gene within the linkage signal on chromosome 7 is *EGFR*. Activation of EGFR is important in the regulation of vascular smooth muscle cells growth, proliferation, and response to injury [47], and has been previously demonstrated to mediate the renin-angiotensin II hypertrophic effect on vascular smooth muscle cells, oxidative stress and interleukin-6 induction, and the chemokine induction of pro-matrix metalloptroteinase2, all leading to AAA [48]. The inhibition of EGFR significantly prevents AAA formation induced by renin-angiotensin II in mice [49]. This region has been previously associated with smoking behavior [50], serum IgG glycosylation [51], and circulating cytokines [52] in GWAS.

While we performed fine-mapping to seek associated SNPs under the linkage signal, we could not find any significant association with the aortic diameter in our sample, or with AAA in the UK-Biobank available data. While acknowledging the fact that our sample size may have limited power to detect association signals, this lack of associations suggests that rare variation could be driving the linkage signal. As described before, GWAS have limited power for the identification of rare variation. A suggested approach for finding the additional genetic components is to focus on low frequency and rare variants [53]. Linkage analyses exploiting family data can have the power to detect rare variants that segregate in affected families, representing a necessary complementary approach to existing GWAS [54]. Despite being rare in the population, rare variants might have a strong impact on disease, and, more importantly, might indicate genes and biological pathways that are relevant to the etiology of the disease. Our results, which are in agreement with previous findings in animal work demonstrating a link between EGFR and AAA, confirm this hypothesis and emphasize the importance to complement GWAS and linkage studies for better coverage of genetic effects.

The second significant linkage signal was located on chromosome 4, close to *BTC* gene. Interestingly, BTC is an endogenous EGFR ligand that has been described to enhance cell migration by binding to EGFR [55], therefore, suggesting that both linkage signals reflect the importance of the same biological pathway affecting aortic diameter and AAA risk through EGFR-mediated transduction of inflammatory signals.

Overlapping our results with other GWAS in the region, we found that these regions had been previously associated with spirometry pulmonary function tests and COPD, which could reflect an effect of EGFR also to mediate cytokine signals within the lung context. Despite previous evidence linking EGFR to AAA, further studies are needed to confirm the role of these genes in healthy abdominal aortic diameter.

In conclusion, we have presented a new study to understand genetic factors regulating the diameter of the aorta in individuals at high-risk of AAA, and to investigate novel variables that can predict AAA before the diameter exceeds pathological limits. While this is the first study presenting the cohort’s baseline characteristics and follow-up studies will be essential for the identification of novel AAA risk factors, the present results are important to establish the main regulators of normal variability in aortic diameter in the “healthy” population and suggest that some genetic AAA risk factors might be detectable even before aortic diameter has exceeded pathological size. In this regard, our results confirm age, sex, and body size as main determinants, and identify, for the first time, pulmonary function and creatinine levels as novel predictors of healthy aortic diameter variation. Moreover, our family design allowed identifying common genetic determinants between aortic diameter, pulmonary function, and anthropometric measures and confirm the implication of the EGFR pathway, which had been described before as relevant for AAA, as a potential new regulator of aortic diameter in healthy high-risk individuals. Further investigations, including follow-up of the TAGA individuals and other prospective studies, will be necessary to properly evaluate how baseline characteristics might influence disease risk and to establish novel biomarkers and risk predictors for AAA.

### 4.3. Strengths and Limitations

It is important to note that heritability reflects the proportion of total variability in an observed trait attributable to additive genetic effects in a given population at a particular point in time [56]. These estimates are, therefore, dependent on sample characteristics and should be taken as a measure to understand to what extend a certain trait is genetically determined, but taking into account estimations may vary between different samples [57].

We are aware of the power restrictions derived from our limited sample size. However, this is the initial publication of an ongoing project, where the addition of new families and follow-up of the existing individuals will represent an invaluable resource not existing today to understand AAA risk. Moreover, beyond the future advantage of understanding the real incidence of AAA in high-risk individuals, the genetic design in extended families has some advantages compared to existing studies on unrelated individuals and allows to detect shared genetic regulatory mechanisms between different phenotypes and the diameter of the aorta, thus providing a better understanding of the mechanisms of disease. A family design also provides additional power to detect rare variation, and the present results have demonstrated that additional genetic findings can be found to complement results that are missed in population studies. Finally, an additional advantage of the use of endophenotypes to study disease is that they can be measured in unaffected individuals and can, therefore, provide insight into unaffected carriers of putatively causal disease variants. We see this design as a strength of the study.

## 5. Highlights

1. This study presents a new family study to understand genetic factors regulating the diameter of the aorta in individuals at high risk of AAA.
2. We identify sex, age, body size, and pulmonary function as predictors of healthy aortic diameter.
3. The study reveals two novel loci linked to aortic diameter, containing EGFR and BTC genes, which have previously been related to AAA, however not found in GWAS.
4. The study emphasizes the importance of family studies to complement population-based GWAS studies.

## Figures and Tables

**Table 1:** Demographic characteristics of the individuals included in the study, by gender.

	Male	Female	*p*-Value
*n*	196	211	-
Age (years)	38.7 ± 22.8	38.4 ± 21.3	0.95
Age range years	2–88	2–85	-
BMI (kg/m^2^)	24.8 (5.8)	24.3 (6.0)	0.395
Smokers	101 (51.5)	77 (36.5)	0.003
Dyslipidemia	47 (24.0)	31 (14.7)	0.024
Chronic hypertension	37 (18.9)	26 (12.3)	0.091
Diabetes	12 (6.1)	12 (5.7)	1
Neoplasia	13 (6.6)	13 (6.2)	1
FEV_1_/FVC < 0.70	24 (12.2)	14 (6.6)	0.076
IHD	5 (2.6)	0	0.025
BVD	1 (0.5)	1 (0.5)	1
AAA	11 (5.6)	0	0.001

Nominal variables are presented as number (%) and continuous variables as mean ± SD. BVD: brain-vascular disease; IHD: Ischemic Heart Disease; FEV_1_: Forced Expiratory Volume in 1 s; FVC: Forced Vital Capacity.

**Table 2:** Arterial diameters in males and females per every 20-year interval.

	Male	Female	*p*-Value	Male Including AAA
0–19 years (*n*)	54	54		
Abdominal aorta	10.9 ± 3.0	10.0 ± 2.5	0.11	
Left femoral	6.7 ± 1.6	6.3 ± 1.5	0.19	
Right femoral	6.6 ± 1.6	6.1 ± 1.5	0.14	
Left popliteal	4.5 ± 1.2	4.4 ± 1.1	0.54	
Right popliteal	4.6 ± 1.2	4.3 ± 1.1	0.23	
20–39 years (*n*)	39	50		
Abdominal aorta	15.8 ± 2.2	13.7 ± 1.3	<0.001	
Left femoral	9.3 ± 1.4	7.8 ± 0.7	<0.001	
Right femoral	9.1 ± 1.4	7.7 ± 0.7	<0.001	
Left popliteal	6.3 ± 0.8	5.3 ± 0.7	<0.001	
Right popliteal	6.3 ± 0.9	5.4 ± 0.7	<0.001	
40–59 years (*n*)	65	70		
Abdominal aorta	16.9 ± 2.0	15.1 ± 2.1	<0.001	
Left femoral	9.9 ± 1.1	8.2 ± 1.3	<0.001	
Right femoral	9.8 ± 1.1	8.3 ± 1.1	<0.001	
Left popliteal	6.7 ± 1.0	5.7 ± 1.0	<0.001	
Right popliteal	6.9 ± 1.0	5.7 ± 1.0	<0.001	
>60 years (*n*)	27	37		27+11
Abdominal aorta	19.6 ± 4.2	15.4 ± 2.5	<0.001	31.4 ± 20.1
Left femoral	10.3 ± 1.6	8.8 ± 1.3	<0.001	10.3 ± 1.5
Right femoral	10.6 ± 1.5	8.8 ± 1.4	<0.001	10.6 ± 1.4
Left popliteal	7.7 ± 1.5	6.0 ± 1.0	<0.001	7.4 ± 1.6
Right popliteal	7.6 ± 1.5	6.2 ± 1.2	<0.001	7.5 ± 1.6

Data are presented as mean ± SD (mm).

**Table 3:** Regression coefficients for the main statistically significant covariates (FDR < 0.01). Except for age and sex, all variables were further adjusted by age, age^2^, and sex. Smoking status did not pass the FDR significance threshold, but it was further included for its known significance in aortic-related diseases.

	Beta	SD	*p*-Value	Probands	*n*
Age (years)	0.23	0.01	1.13 × 10^−52^	12	407
Sex (woman)	−1.81	0.16	1.86 × 10^−^^25^	12	407
Left femoral major diameter (mm)	1.29	0.10	3.1 × 10^−31^	10	404
Right femoral major diameter (mm)	1.19	0.11	1.5 × 10^−23^	10	404
Left popliteal major diameter (mm)	1.33	0.14	2.9 × 10^−^^19^	11	388
Right popliteal major diameter (mm)	1.06	0.14	7.4 × 10^−^^14^	11	404
Height (cm)	0.110	0.01	1.4 × 10^−^^23^	11	388
Weight (kg)	0.10	0.01	9.9 × 10^−^^22^	11	388
FEV_1_ (L)	1.66	0.19	8.1 × 10^−^^17^	11	389
FVC (L)	1.43	0.17	1.4 × 10^−^^16^	11	389
Serum creatinine (mmol/L)	0.09	0.01	5.0 × 10^−^^10^	11	367
Waist circumference (cm)	0.08	0.01	3.4 × 10^−^^9^	10	384
BMI (kg/m^2^)	0.14	0.03	8.2 × 10^−^^6^	11	388
Mean corpuscular volume (MCV) (fL)	0.12	0.03	6.1 × 10^−4^	11	367
Smoker	0.26	0.1	0.015	12	313

FEV_1_: Forced expiratory volume in one second; FVC: Forced vital capacity; BMI: Body mass index.

**Table 4:** Phenotypes with significant genetic correlation with the maximum diameter of the aorta (FDR < 0.05).

h^2^	*p*-Value h^2^	Genetic Correlation	*p*-Value	Phenotypic Correlation	*p*-Value	Environmental Correlation	*p*-Value
0.45	1.4 × 10^−11^	0.81	4.5 × 10^−8^	0.48	1.7 × 10^−22^	0.25	1.1 × 10^−2^
0.36	8.9 × 10^−9^	0.82	6.7 × 10^−7^	0.57	2.4 × 10^−33^	0.43	1.1 × 10^−6^
0.41	5.5 × 10^−12^	0.61	1.5 × 10^−4^	0.50	5.7 × 10^−24^	0.44	4.0 × 10^−7^
0.38	2.5 × 10^−9^	0.63	1.7 × 10^−4^	0.39	3.6 × 10^−15^	0.27	2.1 × 10^−3^
0.30	1.5 × 10^−8^	0.60	4.0 × 10^−4^	0.51	9.9 × 10^−25^	0.47	2.7 × 10^−9^
0.27	6.2 × 10^−6^	0.52	5.7 × 10^−3^	0.43	7.7 × 10^−17^	0.39	8.8 × 10^−7^

h^2^: Heritability.

**Table 5:** Significant loci (LOD score > 3) from linkage analysis. Associated phenotypes in the region extracted from the GWAS catalog are included in the last column.

Chr	cM	Max LOD	Closest Genes	Relevant Associated Phenotypes in the Region (GWAS Catalog)
4	83	3.02	BTC	FEV_1_/FVC ratio (PMID: 30804560) FEV_1_ (PMID:30804560) COPD (PMID:30804561)
7	72–73	3.18	EGFR POM121L12 HAUS6P1	Smoking behavior (PMID:30643258) Serum IgG glycosylation (PMID:23382691) Circulating cytokines (PMID:27989323)

Chr: Chromosome; cM: Centimorgan; COPD: Chronic Obstructive Pulmonary Disease; FEV_1_ Forced Expiratory Volume in the first second.

## Supplementary Materials

The following are available online at https://www.mdpi.com/2077-0383/9/4/1242/s1 Supplementary Methods; Figure S1: Graphic of the aortic diameter by family, Table S1: Characteristics of each family pedigree, Table S2: Summary of all variables that have been analyzed in the TAGA study, Table S3: Bivariate analyses with all considered clinical variables and their effect on abdominal aortic diameter, Table S4: Heritabilities, along with genetic, phenotypic and environmental correlations between all clinical variables and aortic diameter.

## Abbreviations

AAA	Abdominal aortic aneurysm
TAGA	Triple A Genomic Analysis
FVC	Forced vital capacity
FEV_1_	Forced expiratory volume in 1 s
MAF	Minor Allele Frequency
IBD	Identical by descent
cM	Integer centimorgan
SNPs	Single nucleotide polymorphisms
GWAS	Genome-wide association study
COPD	Chronic obstructive pulmonary disease
EGFR	Endothelial Growth Factor Receptor
