# Supplementary material for: The TAGA Study: A Study of Factors Determining Aortic Diameter in Families at High Risk of Abdominal Aortic Aneurysm Reveal Two New Candidate Genes
Source: J Clin Med. 2020 Apr 24;9(4):1242. doi: 10.3390/jcm9041242 (PMC7231034; doi:10.3390/jcm9041242)

SUPPLEMENTARY MATERIALS

The TAGA Study: A Study of Factors Determining Aortic Diameter in Families at High Risk of Abdominal Aortic Aneurysm Reveal Two New Candidate Genes

Olga Peypoch [1,2,3†], Ferran Paüls-Vergés [2,†], Miquel Vázquez-Santiago [2,4], Jaime Dilme [1,2,3], Jose Romero [1,2,3], Jordi Giner [3,5], Vicente Plaza [3,5], Jose Roman Escudero [1,2,3], Jose Manuel Soria [2], Mercedes Camacho [2,6,†] and Maria Sabater-Lleal [2,7,†,*]

TABLE OF CONTENTS

Supplementary methods

Phenotype normalization
Estimation of Multipoint IBD matrix
Non-parametric predictive model

Supplementary Table 1: Characteristics of each family pedigree.

Supplementary Table 2: Summary of all variables that have been analyzed in the TAGA study.

Supplementary Table 3: Bivariate analyses with all considered clinical variables and their effect on abdominal aortic diameter.

Supplementary Table 4: Heritabilities, along with genetic, phenotypic, and environmental correlations between all clinical variables and aortic diameter.

Supplementary Figure 1: Graphic of the aortic diameter by family.

Supplementary Methods

Phenotype normalization

Residuals of the initial model were rank-normalized, and a second regression model adjusting for the same covariates (i.e., covariates, kinship matrix, and ascertainment) was applied to the transformed trait to assess the individual effect of each of the 41 variables on the diameter of the aorta. In order to interpret the results, the beta and standard deviation were multiplied by the median absolute deviation to recover the magnitude of the original units.

Estimation of Multipoint IBD matrix

Multipoint IBD estimators were calculated for each integer centimorgan (cM) at all the autosomes using a window of 4 cM and 70 single nucleotide polymorphisms (SNPs) per estimation.

Imputed genotypes were pruned previous to the IBD estimation according to the following criteria: Mendelian errors (~2%), SNPs with low-quality imputation ($R2<0.8$) and low MAF (<0.05) were excluded. A total of 4.665.320 SNPs were used for the estimation of the IBD matrix. Only the first 7 IBD relations were accounted to avoid bias due to identical by state.

Non-parametric predictive model

We performed a non-parametric model to evaluate what limitations might be expected in the predictions of the aorta diameter (if any). Missing phenotypes were imputed to reduce bias in variable selection caused by a reduced sample size. Multiple imputation of missing data based on random forest was done using missForest R package [28]. The response variable (aortic diameter) was not imputed.

In addition, a search for phenotypX10-phenotype interactions was done following the iterative random forest algorithm method, presented in Kumbier et al. [29], using iRF R package. Only interactions with a stability score recovered 45 times from 50 iterations, with 60 bootstraps replicates each (see [29] for details), were accounted. From these, interactions with a phenotype–phenotype correlation higher than 0.7 were excluded to avoid the risk of type II errors.

Since no assumption of normality was needed, no rank-transformation of the phenotypes was performed prior to analyses. In addition, we did not correct for ascertainment bias, since we wanted to see the accuracy accomplished in both cases and controls. Because the conditional forest does not allow to control for relatedness, and this could produce some unexpected bias on the predictions, the prediction for each individual was made excluding all relatives with kinship $\times 2 \geq 0.5$.

Supplementary Table 1: Characteristics of each family pedigree.

Pedigree	Generations	*n*	Age (years)	Age range	Aorta diameter (mm)
1	4	30 [M:18,F:12]	40 ± 23.5	3–78	16 ± 6
2	4	27 [M:16,F:11]	46 ± 20.4	9–81	17 ± 8.8
3	4	35 [M:17,F:18]	35 ± 23.2	2–76	16 ± 8.3
4	5	88 [M:47,F:41]	35 ± 19.7	2–82	15 ± 7.2
5	4	38 [M:19,F:19]	35 ± 23.5	3–80	14 ± 7.7
6	3	14 [M:8,F:6]	43 ± 22.2	9–80	19 ± 13
7	5	37 [M:18,F:19]	44 ± 22.9	6–88	18 ± 14.3
8	4	26 [M:13,F:13]	42 ± 25.8	2–78	19 ± 13.8
9	5	79 [M:37,F:42]	30 ± 19.6	3–81	14 ± 7.7
10	4	23 [M:9,F:14]	51 ± 18.2	11–78	16 ± 12.6
11	3	27 [M:15,F:12]	46 ± 21.4	3–74	17 ± 7.6
12	3	92 [M:39,F:53]	41 ± 22.3	3–86	16 ± 7.4

M = number of males, F = number of females, age, and aorta diameter have the format: Mean ± SD, range = min–max.

Supplementary Table 2: Summary of all variables that have been analyzed in the TAGA study.

Group	Variable
Demographic variables	Gender, Age, Smoker, family, proband, Hypertension, Diabetes, Dyslipidemia, Neoplasia, Ischemic Heart Disease, Brain-vascular disease.
Anthropometric measures	Height, weight, BMI, Waist circumference.
Ultrasound measures	Abdominal aorta diameter, Left femoral major diameter, Right femoral major diameter, Left popliteal major diameter, Right popliteal major diameter.
Pulmonary function tests	FEV_1, FVC, FEV_1/FVC, FEV_1/FVC < 0.70.
Blood cell measures	White blood cell, Absolute neutrophil, Absolute eosinophil, Absolute basophile, Absolute lymphocyte, Absolute monocyte, Red blood cells, Hemoglobin, Ratio absolute lymphocytes versus platelets count, Mean corpuscular volume, Mean corpuscular hemoglobin, Mean corpuscular hemoglobin concentration, Platelet count, Plateletcrit, Mean platelet volume, Platelet distribution width.
Serum levels	Creatinine serum, Serum albumin, Serum glucose, Total bilirubin, Alanine transaminase, Aspartate transaminase, Glomerular filtration rate, Alkaline phosphatase.

FEV_1 = Forced Expiratory Volume in 1 second; FVC = Forced vital capacity.

Supplementary Table 3: Bivariate analyses with all considered clinical variables and their effect on abdominal aortic diameter

Phenotype	Beta	SD	*p*-value	Probands	Sample size
Sex (woman)	-1.81	0.16	1.9X10-25	12	407
Age (years)	0.23	0.01	1.1 X10-52	12	407
Smoking (smoker)	0.26	0.1	0.015	12	313
Height (cm)	0.11	0.01	1.4 X10-23	11	388
Weight (kg)	0.10	0.01	9.9 X10-22	11	388
BMI (kg/m^2)	0.14	0.03	8.2 X10-06	11	388
Waist circumference (cm)	0.08	0.01	3.4 X10-09	10	384
Hipertension (dic.)	1.37	0.49	4.9 X10-03	12	405
Diabetes (dic.)	0.08	0.65	9.1 X10-01	11	406
Dislipemia (dic.)	0.29	0.41	4.8 X10-01	11	405
Left femoral major diameter (mm)	1.29	0.10	3.1 X10-31	10	404
Right femoral major diameter (mm)	1.19	0.11	1.5 X10-23	10	404
Left popliteal major diameter (mm)	1.33	0.14	2.9 X10-19	11	404
Right popliteal major diameter (mm)	1.06	0.14	7.4 X10-14	11	404
White blood cell (L)	0.03	0.09	7.4 X10-01	11	367
Absolute neutrophile (L)	0.16	0.12	1.8 X10-01	11	367
Absolute eosinophile (L)	-0.29	0.79	7.0 X10-01	11	367

Phenotype	Beta	SD	*p*-value	Probands	Sample size
Absolute basophile (L)	-6.21	7.53	4.1 X10-01	11	363
Absolute lymphocyte (L)	-0.36	0.22	9.3 X10-02	11	367
Absolute monocyte (L)	0.51	0.93	5.8 X10-01	11	367
Red blood cells (L)	-0.59	0.45	1.9 X10-01	11	367
Hemoglobin (g/L)	0.02	0.02	2.5 X10-01	11	367
Ratio absolute lymphocytes versus platelets count	-23.60	50.30	6.4 X10-01	11	367
Platelet count (L)	-0.01	0.00	6.7 X10-02	11	367
Plateletcrit (%)	-4.18	2.96	1.6 X10-01	11	367
Mean platelet volume (fL)	0.15	0.18	4.2 X10-01	11	367
Platelet distribution width (fL)	0.10	0.09	2.6 X10-01	11	365
Mean corpuscular volume (MCV) (fL)	0.12	0.03	6.1 X10-04	11	367
Mean corpuscular hemoglobin (%)	0.26	0.09	4.7 X10-03	11	367
Mean corpuscular hemoglobin concentration (g/L)	-0.01	0.02	6.0 X10-01	11	367
Total bilirubin (µmol/L)	0.04	0.03	1.7 X10-01	11	367
Creatinine serum (mmol/ L)	0.09	0.01	5.0 X10-10	11	367
Serum albumin (mmol/L)	0.06	0.06	3.4X10-01	11	367
Serum glucose (mmol/ L)	-0.07	0.21	7.3X10-01	11	367
Alanine transaminase (ALT or GTP) (U/L)	-0.01	0.01	1.9X10-01	11	367
Aspartate transaminase (AST or GOT) (g/L)	-0.02	0.01	9.8X10-02	11	367

Phenotype	Beta	SD	*p*-value	Probands	Sample size
Alkaline phosphatase (µmol/L)	-0.01	0.00	1.0X10-02	11	367
Glomerular filtration rate	-0.01	0.01	4.3X10-01	11	279
FEV_1 (L)	1.66	0.19	8.1X10-17	11	389
FVC (L)	1.43	0.17	1.4X10-16	11	389
FEV_1/FVC (L)	-0.01	0.02	6.6X10-01	11	389

Supplementary Table 4: Heritabilities, along with genetic, phenotypic and environmental correlations between all clinical variables and aortic diameter.

Phenotype	h^2	*p*-value	Gen r^2	*p*-value	Phen r^2	*p*-value	Env r^2	*p*-value
Smoking (smoker)	0.61	8.8X10-05	0.31	0.04	0.19	0.002	0.08	0.51
Height (cm)	0.30	1.5X10-08	0.60	4.0X10-04	0.51	9.9X10-25	0.47	2.7X10-09
Weight (kg)	0.28	3.4X10-04	0.29	1.6X10-01	0.47	8.2X10-20	0.54	5.3X10-19
BMI (kg/m²)	0.33	6.4X10-06	-0.14	5.0X10-01	0.25	1.1X10-06	0.43	2.9X10-07
Waist circumference (cm)	0.12	7.1X10-02	-0.25	4.4X10-01	0.32	2.4X10-09	0.47	4.4X10-09
Hipertension (dic.)	0.07	4.0X10-01	0.91	5.2X10-01	0.28	1.6X10-03	0.29	2.4X10-02
Diabetes (dic.)	0.31	2.1X10-01	0.87	7.6X10-01	-0.02	8.8X10-01	-0.06	7.3X10-01
Dislipemia (dic.)	0.70	2.9X10-03	-0.18	3.8X10-01	0.06	3.6X10-01	0.39	1.1X10-01
Left femoral major diameter (mm)	0.36	8.9X10-09	0.82	6.7X10-07	0.57	2.4X10-33	0.43	1.1X10-06
Right femoral major diameter (mm)	0.41	5.5X10-12	0.61	1.5X10-04	0.50	5.7X10-24	0.44	4.0X10-07
Left popliteal major diameter (mm)	0.45	1.4X10-11	0.81	4.5X10-08	0.48	1.7X10-22	0.25	1.1X10-02
Right popliteal major diameter (mm)	0.38	2.5X10-09	0.63	1.7X10-04	0.39	3.6X10-15	0.27	2.1X10-03
White blood cell (L)	0.52	2.1X10-06	-0.24	1.9X10-01	-0.04	5.5X10-01	0.12	2.6X10-01
Absolute neutrophile (L)	0.43	6.0X10-06	-0.23	2.0X10-01	0.02	6.9X10-01	0.19	5.8X10-02
Absolute eosinophile (L)	0.49	1.9X10-05	0.16	4.0X10-01	0.02	6.7X10-01	-0.07	5.2X10-01
Absolute basophile (L)	0.43	8.8X10-09	-0.09	6.3X10-01	-0.05	4.0X10-01	-0.02	8.0X10-01
Absolute lymphocyte (L)	0.48	1.0X10-07	-0.16	3.8X10-01	-0.11	4.9X10-02	-0.08	4.0X10-01
Absolute monocyte (L)	0.38	3.2X10-04	-0.28	1.6X10-01	-0.02	7.2X10-01	0.13	2.2X10-01
Red blood cells (L)	0.55	9.8X10-09	0.00	9.9X10-01	-0.05	3.4X10-01	-0.10	3.5X10-01

Phenotype	h^2	*p*-value	Gen r^2	*p*-value	Phen r^2	*p*-value	Env r^2	*p*-value
Hemoglobin (g/L)	0.23	2.5X10-04	-0.08	7.4X10-01	0.04	4.1X10-01	0.09	1.8X10-01
Ratio absolute lymphocytes versus platelets count	0.50	3.0X10-10	-0.24	1.5X10-01	-0.05	3.9X10-01	0.09	3.9X10-01
Platelet count (L)	0.49	2.0X10-07	-0.04	7.7X10-01	-0.10	7.1X10-02	-0.15	5.2X10-02
Plateletcrit (%)	0.48	1.4X10-06	-0.04	8.4X10-01	-0.08	1.7X10-01	-0.11	3.0X10-01
Mean platelet volume (fL)	0.86	8.3X10-24	-0.19	1.8X10-01	0.02	7.6X10-01	0.41	1.2X10-02
Platelet distribution width (fL)	0.77	4.9X10-21	-0.22	1.2X10-01	0.04	4.4X10-01	0.41	1.5X10-03
Mean corpuscular volume (MCV) (fL)	0.57	3.8X10-11	0.02	9.1X10-01	0.14	1.7X10-02	0.23	2.5X10-02
Mean corpuscular hemoglobin (%)	0.46	3.0X10-07	-0.11	5.7X10-01	0.11	5.0X10-02	0.25	1.4X10-02
Mean corpuscular hemoglobin concentration (g/L)	0.45	4.0X10-10	0.00	1.0e+00	-0.02	6.1X10-01	-0.04	7.0X10-01
Total bilirubin (µmol/L)	0.36	2.9X10-05	0.36	5.6X10-02	0.10	8.9X10-02	-0.06	5.5X10-01
Creatinine serum (mmol/l)	0.11	5.1X10-02	-0.05	8.5X10-01	0.33	2.2X10-09	0.45	2.0X10-08
Serum albumin (mmol/l)	0.46	3.1X10-11	-0.03	8.4X10-01	0.04	4.5X10-01	0.09	3.3X10-01
Serum glucose (mmol/l)	0.24	1.0X10-02	-0.16	5.9X10-01	-0.01	8.8X10-01	0.03	6.6X10-01
Alanine transaminase (ALT or GTP) (U/L)	0.21	8.9X10-03	-0.50	5.1X10-02	-0.13	2.1X10-02	0.00	9.6X10-01
Aspartate transaminase (AST or GOT) (g/L)	0.08	1.5X10-01	-0.42	2.5X10-01	-0.15	5.7X10-03	-0.11	1.8X10-01
Alkaline phosphatase (µmol/L)	0.08	1.2X10-01	-0.34	3.6X10-01	-0.17	1.2X10-03	-0.15	6.0X10-02
Glomerular filtration rate	0.90	1.4X10-38	0.04	7.3X10-01	-0.08	1.7X10-01	-0.36	2.6X10-02
FEV_1 (L)	0.27	6.2X10-06	0.52	5.7X10-03	0.43	7.7X10-17	0.39	8.8X10-07
FVC (L)	0.30	3.0X10-07	0.45	1.3X10-02	0.42	1.3X10-15	0.40	1.9X10-10
FEV_1/FVC (L)	0.49	1.8X10-06	0.06	6.6X10-01	0.00	9.5X10-01	-0.05	6.5X10-01

h^2= heritability; Gen r^2= genetic correlation; Phen r^2= Phenotypic correlation; Env r^2= Environmental correlation; P= *p*-value for each parameter.

Supplementary Figure 1: Graphic of the distribution of the aortic diameter per family.

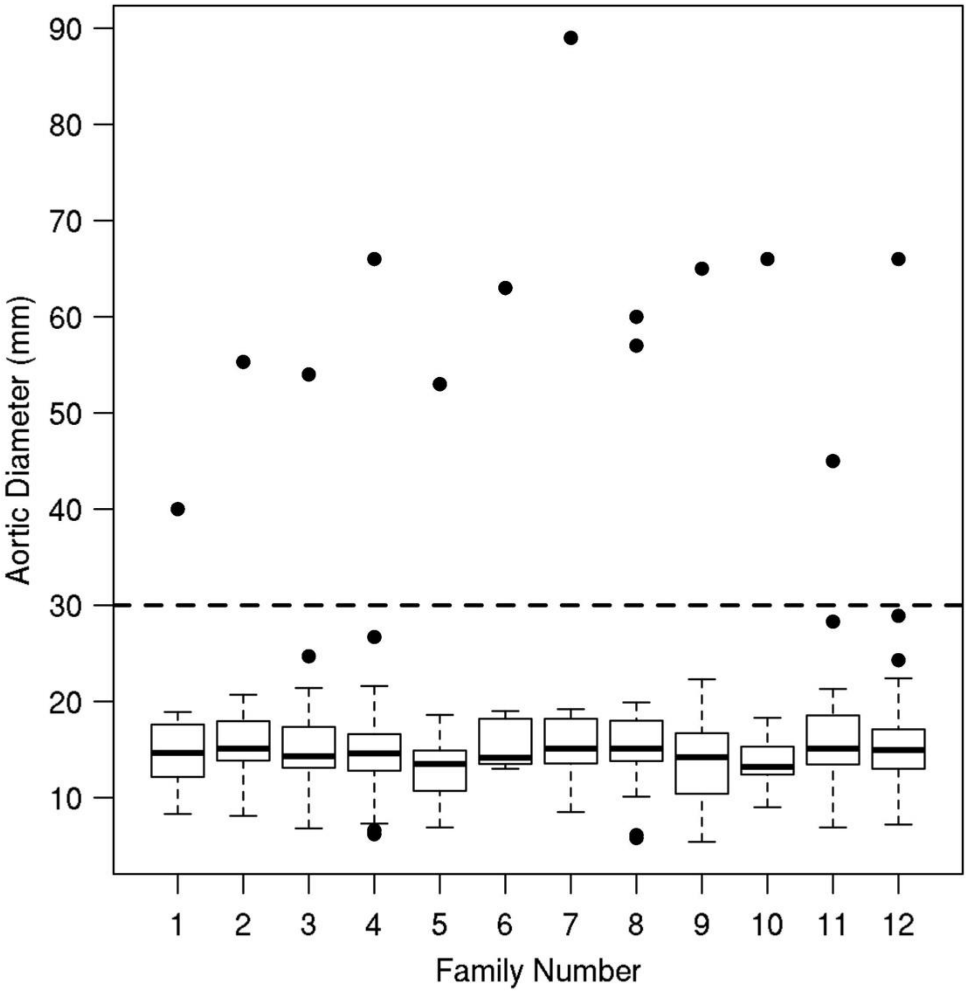

Supplement: Supplementary file 1 [file jcm-09-01242-s001.pdf]
